# Supplementary material for: Effects of pH and Temperature on Water under Pressurized Conditions in the Extraction of Nutraceuticals from Chaga (Inonotus obliquus) Mushroom
Source: Antioxidants (Basel). 2021 Aug 23;10(8):1322. doi: 10.3390/antiox10081322 (PMC8389277; doi:10.3390/antiox10081322)
Supplement: Supplementary file 1 [file antioxidants-10-01322-s001.zip › antioxidants-1310256-supplementary.pdf]

RESULTS

Table S1

Hydrophilic (HAA), Lipophilic (LAA) and total antioxidant (TAA) activities in Chaga (*Inonotus obliquus*) at Various pH values and Temperature treatments

	**Antioxidant activities' values** (µM TE/g Chaga DW)		
**pH and temperature treatment**	**HAA**	**LAA**	**TAA**
pH 2.5 at 60°	106.35± 2.98 [e]	11.36±1.02 [c]	117.72± 3.13 [e]
pH 2.5 at 100°	149.59±2.63 [c]	14.59±0.31 [b]	164.18±2.85 [c]
pH 7 at 60°	92.40±1.35 [f]	9.86±0.06[d]	102.27±1.39 [f]
pH 7 at 100 °	143.36±1.91 [c]	14.09± 0.34 [b]	157.46± 1.97 [c]
pH 9.5 at 60°	119.87± 1.55 [d]	14.65 ± 0.23 [b]	134.52± 1.74 [d]
pH 9.5 at 100°	192.10± 3.30 [b]	12.49± 0.14 [c]	204.59 ± 3.24 [b]
pH 11.5 at 100°	342.85±3.62 [a]	28.01±0.37 [a]	370.86±3.81 [a]

Note: Values (µmol Trolox equivalent/DW g of Chaga extract) represents means ± standard errors. Means that are within the same column and contain different letters indicate a significant difference between the treatments, LSD=0.05, n=4 per each replicate of the sample. HAA= hydrophilic antioxidant activity, LAA= lipophilic antioxidant activity, TAA= total antioxidant activity. Each sample is at a different pH (2.5, 7, 9.5, and 11.5) and temperature (60 ° and 100 °).

Table S2

Hydrophilic (HPC), Lipophilic (LPC)and total phenolic content (TPC) in Chaga (*Inonotus obliquus*) at various pH values and Temperature treatments

	Phenolic Content (µM QE/per g Chaga)		
pH and temperature treatment	HPC	LPC	TPC
pH 2.5 at 60 °	73.83±3.30 [bc]	57.13±1.97 [a]	130.95±4.18 [b]
pH 2.5 at 100°	67.04±4.57 [c]	53.71±0.54 [ab]	120.75±4.24 [b]
pH 7 at 60°	56.00±5.12 [d]	52.75±0.90 [b]	108.32±4.83 [c]
pH 7 at 100°	69.79±2.62 [bc]	52.83±1.62 [b]	122.62±2.14 [b]
pH 9.5 at 60 °	79.50±2.11 [b]	48.05±0.31 [c]	127.56±2.32 [b]
pH 9.5 at 100 °	55.22±3.69 [d]	48.21±1.95 [c]	103.43±4.48 [c]
pH 11.5 at 100°	224.38±2.60 [a]	48.67±0.68 [c]	273.06±2.19 [a]

Note: Values (µmol Quercetin equivalent/ DW g of Chaga extract) represents means ± standard errors. Means that are within the same column and contain different letters indicate a significant difference between the treatments, LSD=0.05, n=4 per each replicate of the sample. HPC= hydrophilic phenolic content, LPC= lipophilic phenolic content, TPC= total phenolic content. Each sample is at a different pH (2.5, 7, 9.5, 11.5) and temperature (60°C and 100°C).

Table S3.A. p-values (Pearson)

Variables	DHTA	DHBA	SalA	ToHBen	DHBA2	MEA	ToPA's	CA	MCA	ToCA	HAA	LAA	TAA	HPC	LPC	TPC
**DHTA**	**0**	**<0.0001**	0.095	**<0.0001**	**<0.0001**	**<0.0001**	**<0.0001**	**<0.0001**	**<0.0001**	**<0.0001**	**0.004**	0.057	**0.004**	0.182	**0.048**	0.222
**DHBA**	**<0.0001**	**0**	**0.000**	**<0.0001**	**<0.0001**	**0.000**	**<0.0001**	**<0.0001**	**<0.0001**	**<0.0001**	**<0.0001**	**0.001**	**<0.0001**	**0.002**	**0.013**	**0.004**
**SalA**	0.095	**0.000**	**0**	**0.003**	0.063	**0.029**	0.062	**0.030**	0.063	0.055	**<0.0001**	**<0.0001**	**<0.0001**	**<0.0001**	0.061	**<0.0001**
**ToHBen**	**<0.0001**	**<0.0001**	**0.003**	**0**	**<0.0001**	**<0.0001**	**<0.0001**	**<0.0001**	**<0.0001**	**<0.0001**	**<0.0001**	**0.002**	**<0.0001**	**0.009**	**0.019**	**0.012**
**DHBA2**	**<0.0001**	**<0.0001**	0.063	**<0.0001**	**0**	**<0.0001**	**<0.0001**	**<0.0001**	**<0.0001**	**<0.0001**	**0.001**	**0.019**	**0.001**	0.073	0.129	0.085
**MEA**	**<0.0001**	**0.000**	**0.029**	**<0.0001**	**<0.0001**	**0**	**<0.0001**	**<0.0001**	**<0.0001**	**<0.0001**	**0.004**	0.064	**0.004**	0.112	0.115	0.138
**ToPA's**	**<0.0001**	**<0.0001**	0.062	**<0.0001**	**<0.0001**	**<0.0001**	**0**	**<0.0001**	**<0.0001**	**<0.0001**	**0.001**	**0.019**	**0.001**	0.074	0.129	0.086
**CA**	**<0.0001**	**<0.0001**	**0.030**	**<0.0001**	**<0.0001**	**<0.0001**	**<0.0001**	**0**	**<0.0001**	**<0.0001**	**0.000**	**0.009**	**0.000**	**0.045**	0.092	0.053
**MCA**	**<0.0001**	**<0.0001**	0.063	**<0.0001**	**<0.0001**	**<0.0001**	**<0.0001**	**<0.0001**	**0**	**<0.0001**	**0.001**	**0.019**	**0.001**	0.073	0.129	0.085
**ToCA**	**<0.0001**	**<0.0001**	0.055	**<0.0001**	**<0.0001**	**<0.0001**	**<0.0001**	**<0.0001**	**<0.0001**	**0**	**0.001**	**0.016**	**0.001**	0.067	0.121	0.078
**HAA**	**0.004**	**<0.0001**	**<0.0001**	**<0.0001**	**0.001**	**0.004**	**0.001**	**0.000**	**0.001**	**0.001**	**0**	**<0.0001**	**<0.0001**	**<0.0001**	**0.050**	**<0.0001**
**LAA**	0.057	**0.001**	**<0.0001**	**0.002**	**0.019**	0.064	**0.019**	**0.009**	**0.019**	**0.016**	**<0.0001**	**0**	**<0.0001**	**<0.0001**	0.167	**<0.0001**
**TAA**	**0.004**	**<0.0001**	**<0.0001**	**<0.0001**	**0.001**	**0.004**	**0.001**	**0.000**	**0.001**	**0.001**	**<0.0001**	**<0.0001**	**0**	**<0.0001**	0.055	**<0.0001**
**HPC**	0.182	**0.002**	**<0.0001**	**0.009**	0.073	0.112	0.074	**0.045**	0.073	0.067	**<0.0001**	**<0.0001**	**<0.0001**	**0**	0.301	**<0.0001**
**LPC**	**0.048**	**0.013**	0.061	**0.019**	0.129	0.115	0.129	0.092	0.129	0.121	**0.050**	0.167	0.055	0.301	**0**	0.498
**TPC**	0.222	**0.004**	**<0.0001**	**0.012**	0.085	0.138	0.086	0.053	0.085	0.078	**<0.0001**	**<0.0001**	**<0.0001**	**<0.0001**	0.498	**0**

Table S3.B. Coefficients of determination (Pearson)

Variables	DHTA	DHBA1	SalA	ToHBen	DHBA2	MEA	ToPA's	CA	MCA	ToCA	HAA	LAA	TAA	HPC	LPC	TPC
**DHTA**	**1**	0.609	0.140	0.776	0.851	0.652	0.853	0.896	0.850	0.867	0.365	0.178	0.357	0.092	0.191	0.077
**DHBA**	0.609	**1**	0.491	0.936	0.647	0.535	0.647	0.704	0.648	0.662	0.797	0.460	0.783	0.399	0.282	0.367
**SalA**	0.140	0.491	**1**	0.372	0.171	0.228	0.171	0.225	0.171	0.180	0.796	0.785	0.798	0.820	0.173	0.780
**ToHBen**	0.776	0.936	0.372	**1**	0.781	0.573	0.781	0.862	0.781	0.802	0.697	0.410	0.687	0.311	0.259	0.288
**DHBA2**	0.851	0.647	0.171	0.781	**1**	0.739	1.000	0.940	1.000	0.998	0.447	0.258	0.442	0.159	0.117	0.148
**MEA**	0.652	0.535	0.228	0.573	0.739	**1**	0.740	0.717	0.739	0.741	0.364	0.169	0.358	0.128	0.125	0.112
**ToPA's**	0.853	0.647	0.171	0.781	1.000	0.740	**1**	0.941	1.000	0.998	0.446	0.258	0.442	0.159	0.117	0.147
**CA**	0.896	0.704	0.225	0.862	0.940	0.717	0.941	**1**	0.940	0.959	0.495	0.305	0.492	0.195	0.142	0.184
**MCA**	0.850	0.648	0.171	0.781	1.000	0.739	1.000	0.940	**1**	0.998	0.447	0.258	0.443	0.159	0.117	0.148
**ToCA**	0.867	0.662	0.180	0.802	0.998	0.741	0.998	0.959	0.998	**1**	0.458	0.267	0.454	0.166	0.122	0.154
**HAA**	0.365	0.797	0.796	0.697	0.447	0.364	0.446	0.495	0.447	0.458	**1**	0.847	0.999	0.813	0.188	0.783
**LAA**	0.178	0.460	0.785	0.410	0.258	0.169	0.258	0.305	0.258	0.267	0.847	**1**	0.862	0.952	0.098	0.936
**TAA**	0.357	0.783	0.798	0.687	0.442	0.358	0.442	0.492	0.443	0.454	0.999	0.862	**1**	0.827	0.180	0.798
**HPC**	0.092	0.399	0.820	0.311	0.159	0.128	0.159	0.195	0.159	0.166	0.813	0.952	0.827	**1**	0.056	0.989
**LPC**	0.191	0.282	0.173	0.259	0.117	0.125	0.117	0.142	0.117	0.122	0.188	0.098	0.180	0.056	**1**	0.024
**TPC**	0.077	0.367	0.780	0.288	0.148	0.112	0.147	0.184	0.148	0.154	0.783	0.936	0.798	0.989	0.024	**1**
